# Prevalence and predictors of coronary heart disease among individuals with and without obesity in North Dakota

**DOI:** 10.1371/journal.pone.0313283

**Published:** 2024-11-08

**Authors:** Emmanuel Angmorteh Mensah, Md Marufuzzaman Khan, Agricola Odoi, Grace Njau, Matthew Schmidt, Jennifer Lord

**Affiliations:** 1 Department of Biostatistics & Epidemiology, College of Public Health, East Tennessee State University, Johnson City, TN, United States of America; 2 Beth Israel Deaconess Medical Center, Boston, MA, United States of America; 3 Department of Biomedical and Diagnostic Sciences, College of Veterinary Medicine, University of Tennessee, Knoxville, TN, United States of America; 4 North Dakota Department of Health and Human Services, Special Projects and Health Analytics, Bismarck, ND, United States of America; Kurume University School of Medicine, JAPAN

## Abstract

**Introduction:**

Coronary heart disease (CHD) is the most common cardiovascular disease in the United States and heart disease is the leading cause of death in North Dakota. Although obesity is a major risk factor for CHD, some studies have reported an inverse relationship between body mass index (BMI) and CHD outcomes. Understanding the epidemiology of CHD among individuals with and without obesity is essential to guide health planning. Therefore, the objectives of this study were to estimate the prevalence and identify predictors of CHD among North Dakota adults with and without obesity.

**Methods:**

Behavioral Risk Factor Surveillance System data from 2017 to 2021 were provided by the North Dakota Department of Health and Human Services. Weighted prevalence estimates and 95% confidence intervals (CIs) were computed for CHD and potential risk factors among respondents with and without obesity (BMI ≥30 kg/m^2^). Multivariable logistic regression was used to identify significant predictors of CHD in both groups.

**Results:**

A total of 17,092 respondents were included in the study. Respondents with obesity had a higher prevalence of CHD (4.7%, 95% CI: 4.2–5.4) than those without obesity (3.0%, 95% CI: 2.7–3.4). Predictors of CHD among both groups included age, sex, self-reported general health, high blood pressure, high cholesterol, chronic obstructive pulmonary disease, and diabetes. Having a personal doctor (Odds Ratio [OR] = 1.91, 95% CI: 1.13–3.23) and arthritis (OR = 1.72, 95% CI: 1.34–2.22) were significant predictors of CHD among respondents without obesity, but not among those without obesity.

**Conclusion:**

While the prevalence of CHD was significantly higher among adults with obesity compared to those without obesity, the findings of the stratified analysis indicated that predictors of the condition tended to be similar for the two groups. This study provides useful information to guide health planning and control programs that aim to reduce the burden of CHD in North Dakota.

## Introduction

Cardiovascular disease (CVD) is the leading cause of mortality worldwide and presents in many forms, including congenital heart disease, deep vein thrombosis, cerebrovascular disease, coronary heart disease, peripheral arterial disease, and rheumatic heart illnesses [1, 2]. However, coronary heart disease (CHD) is the most common subtype in Europe and North America [3, 4]. The condition occurs due to the accumulation of cholesterol, fat, and calcium deposits in the arteries, leading to arterial constriction, stiffening and decreased oxygen supply to the heart [5]. Coronary heart disease may manifest as chest pain (angina), heart failure, heart attack, and irregular heartbeats (arrhythmia) [5].

Approximately 244.1 million individuals are living with heart disease globally [6]. The global CHD mortality rate was estimated to be 112.37 per 100,000 cases in 2022 [6]. In the United States, approximately 366,000 coronary heart disease deaths occur each year [1], and 1 in 20 adults who are 20 years and older have CHD [7]. Coronary heart disease is a leading cause of morbidity and mortality across all states in the US, including North Dakota [8]. The condition is also associated with a significant financial burden. In 2015, CHD was associated with $89 billion in direct costs in the United States, and this is projected to increase to $215 billion by 2035 [3]. Furthermore, it was estimated that heart disease resulted in $203.3 billion in income losses in 2018 [9]. At the individual level, CHD is associated with poor physical health and diminished functional capacity, and those with CHD experience lengthy periods of sleep, depression, and tiredness [10, 11]. Furthermore, research has linked coronary heart disease to higher hospital readmission rates [12].

Obesity is an important risk factor for CHD, and approximately 80% of CHD patients are either overweight or have obesity [13, 14]. Compared to individuals with normal weight, those with obesity have significantly greater incidence of pre-clinical atherosclerosis [15]. Furthermore, the risk of coronary heart disease is 1.27 times higher for every 5 kg/m^2^ increase in body mass index (BMI) [16]. However, it is worth noting that some studies have reported an inverse relationship between obesity and CHD outcomes, a phenomenon that has been observed for numerous cardiovascular diseases and has been termed the “obesity paradox” [17–20]. Such studies have reported that patients with cardiovascular disease who have a diagnosis of overweight or obesity based on body mass index have a better prognosis than those whose BMI is classified as normal or underweight [19–21]. Several explanations for this paradox have been proposed, including earlier diagnosis of cardiovascular disease and more frequent screening among those with obesity [17, 20], but there is limited research investigating whether characteristics associated with CHD differ for those with and without obesity. Investigating determinants of CHD among individuals with and without obesity is important to better understand the epidemiology of the disease and gather information to guide control programs. This is especially critical for the state of North Dakota because heart disease is the leading cause of deaths in this state, which has an estimated heart disease prevalence of 152.8 per 100,000 [8]. Therefore, the objectives of this study were to estimate the prevalence and identify predictors of coronary heart disease among North Dakota adults with and without obesity.

## Methods

### Ethics approval

This study was approved by the University of Tennessee Institutional Review Board (Number: UTK IRB-24-08175-XM). The study used secondary data provided to the investigators by the North Dakota Department of Health and Human Services. The need for informed consent was waived by the Institutional Review Board. Study investigators did not have information that could be used to identify individual participants. All data were fully anonymized before being shared with the study investigators.

### Study area and design

This retrospective cross-sectional study used survey data from the Behavioral Risk Factor Surveillance System (BRFSS) to investigate socioeconomic, demographic, and lifestyle factors as potential predictors of coronary heart disease (CHD) among individuals with and without obesity in North Dakota. North Dakota is comprised of 53 counties, six of which are designated as urban and ten as semi-rural, with the remainder classified as rural [22]. Urban counties contain a metro area with a population of 50,000 or greater, while those classified as semi-rural contain a town or city with a population between 2,500 and 49,999. Counties that do not contain a town or city with a population of 2,500 or greater are classified as rural [23]. As of 2022, North Dakota had an estimated population of 779,261 residents, 48.6% of whom were female [24]. Persons under the age of 18 represented 23.5% of the population, and 16.7% were aged 65 years and older [25]. The majority of the population was white (86.6%). The next largest racial groups were American Indian or Alaskan Native (5.3%) and Black or African American (3.6%). Other races and ethnic minorities represent a minuscule proportion of North Dakota’s total population [24, 25].

### Data sources and variable selection

**Fig 1:**
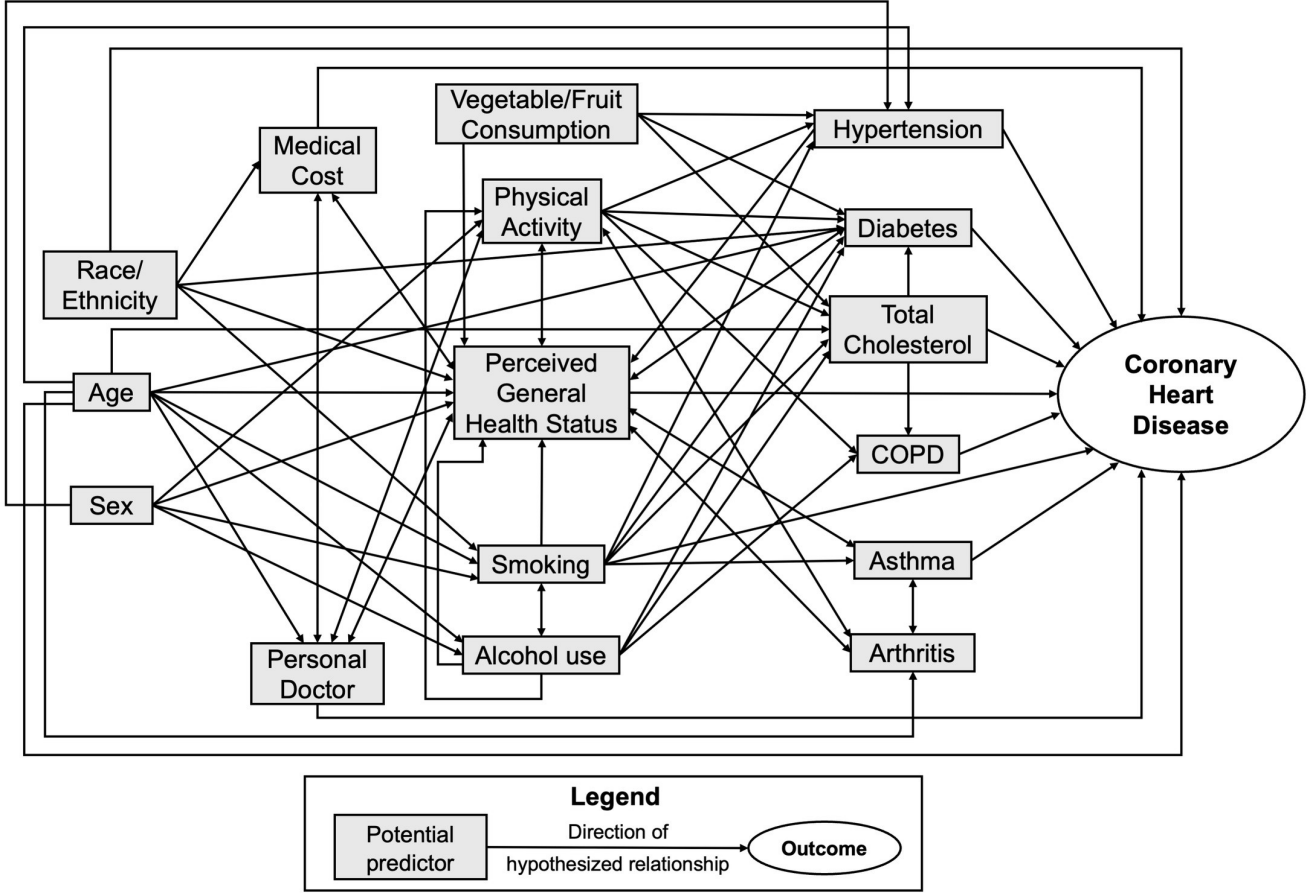
Conceptual model depicting potential predictors of coronary heart disease among respondents to the North Dakota Behavioral Risk Factor Surveillance System survey.

Behavioral Risk Factor Surveillance System (BRFSS) data from 2017 to 2021 were provided to the investigators by the North Dakota Department of Health and Human Services (NDDOHHS). The BRFSS is a nationwide survey conducted by state health departments with assistance from the Centers for Disease Control and Prevention (CDC) that gathers data on health-related risk behaviors, chronic health conditions and use of preventive services [26]. The study population included adults aged 18 years and older. A respondent with CHD was defined as “a person who has ever been informed by health workers that they have angina or coronary heart disease”. To guide the selection of variables from the dataset for inclusion in the study as potential predictors of CHD, a conceptual model was built (Fig 1). The conceptual model included variables that were available in the BRFSS dataset and were hypothesized to have biological, physical, and/or social relationships with CHD based on evidence from the existing literature [27]. These variables are defined in Table 1. Since some variables of interest were not available during all years of the study period, only records from 2017, 2019 and 2021 were included in the study.

**Table 1:** List and definitions of Behavioral Risk Factor Surveillance System variables considered as potential predictors of coronary heart disease among North Dakota adults.

Variable	Definition
Sex	Sex of respondent
Age	Age of respondent
Race	Race and ethnicity of respondent
General health	Perceived health status of respondent
Personal doctor	Having one or more doctors as personal healthcare providers
Medical cost	Respondent could not afford to consult a doctor when needed in the past 12 months
Physical activity	Respondent reported performing any form of physical activity in the past 30 days
Current smoker	Respondent currently smokes and has smoked at least 100 cigarettes in the past
Heavy drinker	Consumption of 14 or more alcoholic drinks per week (males) or 7 or more alcoholic drinks per week (females)
Fruit consumption	Consumption of fruit one or more times per day
Vegetable consumption	Consumption of vegetables one or more times per day
High blood pressure	Respondent notified by healthcare professional that they have high blood pressure
High cholesterol	Respondent notified by healthcare professional that they have high cholesterol
Arthritis	Respondent notified by healthcare professional that they have arthritis
COPD	Respondent notified by healthcare professional that they have chronic obstructive pulmonary disease (COPD)
Asthma	Respondent notified by healthcare professional that they have asthma
Diabetes	Respondent notified by healthcare professional that they have diabetes

### Data preparation

Respondent age was classified into three categories (18–44, 45–64, and 65 years and older). Since racial and ethnic minorities represent a small fraction of the population in North Dakota, respondent race and ethnicity were classified into one of the following four categories: Non-Hispanic White, Non-Hispanic American Indian (A. Indian)/Alaskan Native, Hispanic, or multiracial or other non-Hispanic. Respondents were considered to have obesity if their body mass index (BMI) was ≥ 30 kg/m^2^. Individuals without obesity included those classified as normal weight (BMI ≥ 18.50 - < 25.00), underweight (BMI < 18.50) or overweight (BMI ≥ 25.00 - < 30.00).

### Statistical analysis

Statistical analysis was performed using STATA 17.0 [28]. To account for the complex survey design used for data collection in the BRFSS survey, the sampling weight (_LLCPWT), primary sampling unit (_PSU) and strata (_STSTR) variables were applied when computing frequencies and conducting statistical analyses to ensure that estimates were representative of the North Dakota population [29]. Statistical analyses were conducted separately for respondents with obesity (*n* = 6,021) and those without obesity (*n* = 11,071). Unweighted frequencies and weighted percentages and 95% confidence intervals (CIs) of the percentages were computed for all categorical variables.

Multivariable logistic regression was used to identify significant predictors of CHD among North Dakota BRFSS respondents. To identify candidate variables for inclusion in the model-building process, univariable associations between CHD and each of the potential predictor variables from the conceptual model in Fig 1 were first assessed using survey logistic regression. Variables that had significant univariable associations with CHD at a *p*-value of ≤ 0.2 were considered for inclusion in multivariable models. To prevent problems with multicollinearity in the multivariable models, correlations between all possible pairs of potential predictors were assessed using Spearman correlation tests. Only one of a pair of highly correlated variables (|*r*_*s*_| ≥ 0.7) was retained for further analysis. However, no pairs of highly correlated variables were identified among the potential predictors.

Multivariable logistic regression models were built using manual backward elimination, specifying a critical *p*-value of 0.05 for elimination of variables. Confounding was evaluated by assessing changes in regression coefficients following the removal of each variable during the backward elimination process. A change of 20% or more in the magnitude of the regression coefficient(s) of one or more other variables in the model following the removal of a variable indicated potential confounding. Confounding variables were retained in the model regardless of statistical significance. Biologically meaningful two-way interaction terms, based on literature review, were assessed for statistical significance using a critical *p*-value of 0.05. The goodness of fit of the final models was assessed using the Hosmer-Lemeshow goodness-of-fit test.

## Results

### Descriptive analyses

A total of 17,092 respondents to the North Dakota BRFSS survey were included in the study, 35.4% of whom had obesity. A significantly higher percentage of respondents with obesity were male (55.7%) compared to those without obesity (51.6%) (Table 2). Among those with obesity, there was a lower percentage of respondents between the ages of 18 and 44 (45.3%) compared to those without obesity (52.2%). The vast majority of respondents in both groups were non-Hispanic White. A higher percentage of respondents without obesity considered themselves to be in good health (89.4%) compared to those with obesity (80.0%). While the percentage of respondents that reported experiencing cost barriers to obtaining medical care was similar between the two groups, a higher percentage of those with obesity reported having a personal doctor (79.3%) compared to those without obesity (71.5%). Respondents with obesity had a higher prevalence of coronary heart disease (4.7%, 95% Confidence Interval [CI]: 4.2–5.4) compared to those without obesity (3.0%, 95% CI: 2.7–3.4).

**Table 2:** Sociodemographic characteristics, health behaviors and chronic conditions among BRFSS respondents with and without obesity (BMI ≥ 30 kg/m^2^) in North Dakota (2017, 2019, and 2021).

Characteristics	Categories	BMI^1^ < 30 (n = 11,071)		BMI^1^ ≥ 30 (n = 6,021)	
		n	Weighted % (95% CI^2^)	n	Weighted % (95% CI^2^)
Sex	Male	5,351	51.6 (50.3, 52.9)	3,221	55.7 (53.9, 57.4)
	Female	5,717	48.4 (47.1, 49.7)	2,800	44.4 (42.6, 46.1)
Age	18–44	2,880	52.2 (51.0, 53.5)	1,397	45.3 (43.6, 47.1)
	45–64	3452	26.6 (25.6, 27.7)	2,283	34.15 (32.6, 35.7)
	65+	4,647	21.2 (20.4, 21.9)	2,315	20.6 (19.6, 21.6)
Race	White	10,149	86.7 (85.6, 87.8)	5,392	85.2 (83.7, 86.6)
	A.Indian/Alaska Native	314	3.2 (2.8, 3.7)	341	6.6 (5.7, 7.5)
	Hispanic	134	3.2 (2.6, 3.8)	86	3.7 (2.9, 4.7)
	Others/multiracial	375	6.9 (6.1, 7.8)	161	4.5 (3.7, 5.4)
General Health	Good	9,556	89.4 (88.6, 90.1)	4,733	80.0 (78.6, 81.4)
	Poor/Fair	1503	10.6 (9.9, 11.4)	1,282	20.0 (18.6, 21.4)
Personal Doctor	Yes	8,823	71.5 (70.2, 72.8)	5,125	79.3 (77.8, 80.8)
	No	2180	28.5 (27.3, 29.8)	872	20.7 (19.2, 22.2)
Medical Cost	Could see a Doctor	10,503	92.6 (91.8, 93.4)	5,650	91.2 (90.0, 92.3)
	Could not see Doctor	541	7.4 (6.6, 8.20)	355	8.8 (7.7, 10.0)
Physical Activity	Yes	8,100	76.5 (75.4, 77.6)	3,921	66.9 (65.3, 68.6)
	No	2,757	23.5 (22.4, 24.6)	2,010	33.1 (31.4, 34.7)
Current Smoker	Yes	1,639	17.2 (16.2, 18.3)	761	17.1 (15.7, 18.6)
	No	9,290	82.8 (81.7, 83.8)	5,196	82.9 (81.5, 84.3)
Heavy Drinker	Yes	637	7.2 (6.5, 8.0)	337	7.6 (6.6, 8.8)
	No	10,097	92.8 (92.0, 93.5)	5,514	92.4 (91.2, 93.4)
Fruit	1 or more/day	6,852	61.8 (60.5, 63.1)	3,434	54.3 (52.5, 56.1)
Consumption	Less than 1/day	3,636	38.2 (36.9, 39.5)	2,308	45.7 (39.3, 47.5)
Vegetable	1 or more/day	8,386	78.8 (77.7, 80.0)	4,496	78.0 (76.4, 79.4)
Consumption	Less than 1/day	1,922	21.2 (20.0, 22.3)	1,181	22.0 (20.6, 23.6)
High Blood	Yes	3,854	23.6 (22.7, 24.6)	3,230	43.0 (41.4, 44.7)
Pressure	No	7,192	76.4 (75.4, 77.3)	2,781	57.0 (55.3, 58.6)
High	Yes	3,390	28.0 (26.9, 29.1)	2,461	37.3 (35.6, 39.0)
Cholesterol	No	5,968	72.0 (70.9, 73.1)	3,002	62.7 (61.0, 64.4)
Arthritis	Yes	3,382	21.0 (20.1, 22.0)	2,542	33.1 (31.6, 34.6)
	No	7,646	79.0 (78.1, 79.9)	3,454	66.9 (65.4, 68.5)
COPD^3^	Yes	691	4.2 (3.8, 4.7)	458	6.2 (5.5, 7.0)
	No	10,335	95.8 (95.3, 96.2)	5,535	93.8 (93.0, 94.5)
Asthma	Yes	1,058	11.0 (10.2, 11.9)	791	15.3 (14.0, 16.7)
	No	9,985	89.0 (88.1, 89.8)	5,209	84.8 (83.4, 86.0)
Diabetes	Yes	949	5.6 (5.1, 6.1)	1,240	15.7 (14.7, 16.9)
	No	10,114	94.4 (93.9, 94.9)	4,778	84.3 (83.2, 85.4)
Coronary heart disease	Yes	573	3.0 (2.7, 3.4)	417	4.7 (4.2, 5.4)
	No	10,405	97.0 (96.6, 97.3)	5,544	95.3 (94.6, 95.8)

^1^Body mass index,

^2^Confidence interval,

^3^Chronic obstructive pulmonary disease

The percentage of respondents who reported that they did not engage in regular physical activity was higher among those with obesity (33.1%) than those without obesity (23.5%). Levels of cigarette smoking and heavy alcohol consumption did not differ substantially between respondents with obesity and those without obesity. While fruit consumption tended to be more frequent among respondents without obesity, vegetable consumption was similar between the two groups. The percentage of respondents who reported eating fruit one or more times per day was 61.8% among those without obesity, compared to 54.3% of those with obesity. The percentage of respondents who reported eating vegetables at least once per day was 78.8% among respondents without obesity and 78.0% among those with obesity.

Comorbidities tended to be more common among those with obesity compared to those without obesity. For instance, 43.0% of respondents with obesity had hypertension and 15.7% had diabetes, compared to 23.8% and 5.6% of respondents without obesity, respectively. High cholesterol (37.3% vs. 28.0%), arthritis (33.1% vs. 21.0%), chronic obstructive pulmonary disease (COPD, 6.2% vs. 4.2%), and asthma (15.3% vs. 11.0%) were also more common among respondents with obesity.

### Predictors of coronary heart disease among respondents with and without obesity

A number of variables had significant univariable associations with coronary heart disease among individuals with and without obesity (Table 3). Results of the two final multivariable logistic regression models, one for respondents with obesity and the other for those without, are displayed in Table 4. Predictors of coronary heart disease in both models included sex, age, self-reported general health status, high blood pressure, high cholesterol, COPD, and diabetes (Table 4). Among both groups, the odds of coronary heart disease among females were approximately 50% lower than the odds of CHD among males. Older age was also a significant predictor of coronary heart disease regardless of whether a respondent had obesity. Among those without obesity, the odds of CHD were higher among individuals between the ages of 45 and 64 (Odds Ratio [OR] = 3.16, 95% CI: 1.28–7.80) and those aged 65 and over (OR = 9.49, 95% CI: 3.81–23.6) compared to those between the ages of 18 and 44. Among respondents with obesity, those aged 65 and over also had significantly higher odds of CHD compared to those between the ages of 18 and 44 (OR = 5.42, 95% CI: 2.02–14.5), but the association was not significant for those between the ages of 45 and 64.

**Table 3:** Univariable associations between coronary heart disease and selected potential predictors among BRFSS respondents with and without obesity (BMI ≥ 30 kg/m^2^) in North Dakota (2017, 2019, and 2021).

Characteristics	Categories	BMI^1^ < 30 (*n* = 11,071)		BMI^1^ ≥ 30 (*n* = 6,021)	
		Unadjusted Odds Ratio	*p*-value	Unadjusted Odds Ratio	*p*-value
		(95% CI ^2^)		(95% CI^2^)	
Sex	Female	0.67 (0.53, 0.83)	< 0.001	0.62 (0.47, 0.81)	0.001
	Male	Reference		Reference	
Age			< 0.001		< 0.001
	65+	42.33 (19.83, 90.38)	< 0.001	15.41 (6.76, 35.13)	< 0.001
	45–64	9.44 (4.29, 20.79)	< 0.001	5.21 (2.25, 12.10)	< 0.001
	18–44	Reference		Reference	
Race/ethnicity			0.0001		0.894
	A.Indian/Alaska Native	1.06 (0.60, 0.84)	0.840	1.09 (0.62, 1.90)	0.768
	Hispanic	0.10 (0.01, 0.73)	0.023	0.49 (0.07, 3.56)	0.481
	Others/multiracial	0.26 (0.12, 0.57)	0.001	1.09 (0.35, 3.37)	0.883
	Non-Hispanic White	Reference		Reference	
General Health	Poor/Fair	7.30 (5.81, 9.17)	< 0.001	4.34 (3.28, 575)	< 0.001
	Good	Reference		Reference	
Personal Doctor	Yes	5.60 (3.57, 8.78)	< 0.001	3.59 (1.79, 7.19)	< 0.001
	No	Reference		Reference	
Medical Cost	Could see a doctor	1.41 (0.83, 2.39)	0.207	1.11 (0.52, 2.34)	0.790
	Could not see doctor	Reference		Reference	
Physical Activity	Yes	0.67 (0.53, 0.85)	0.001	0.60 (0.45, 0.79)	< 0.001
	No	Reference		Reference	
Current Smoker	Yes	1.02 (0.76, 1.39)	0.884	1.03 (0.65, 1.63)	0.895
	No	Reference		Reference	
Heavy Drinker	Yes	0.42 (0.22, 0.80)	0.009	0.44 (0.11, 1.78)	0.249
	No	Reference		Reference	
Fruit	1 or more/day	1.30 (1.04, 1.64)	0.024	1.24 (0.92, 1.67)	0.151
Consumption	Less than 1/day	Reference		Reference	
Vegetable	1 or more/day	0.91 (0.69, 1.19)	0.480	1.27 (0.91, 1.78)	0.156
Consumption	Less than 1/day	Reference		Reference	
High Blood	Yes	8.40 (6.62, 10.65)	< 0.001	8.57 (5.82, 12.62)	< 0.001
Pressure	No	Reference		Reference	
High Cholesterol	Yes	4.91 (3.88, 6.21)	< 0.001	4.87 (3.61, 6. 57)	< 0.001
	No	Reference		Reference	
Arthritis	Yes	5.04 (4.06, 6.26)	< 0.001	3.63 (2.76, 4.77)	< 0.001
	No	Reference		Reference	
COPD^3^	Yes	6.39 (4.79, 8.53)	< 0.001	5.27 (3.61, 7.70)	< 0.001
	No	Reference		Reference	
Asthma	Yes	1.00 (0.71, 1.40)	0.979	1.26 (0.82, 1.95)	0.293
	No	Reference		Reference	
Diabetes	Yes	6.18 (4.75, 8.05)	< 0.001	5.42 (4.10, 7.18)	< 0.001
	No	Reference		Reference	

^1^Body mass index,

^2^Confidence interval,

^3^Chronic obstructive pulmonary disease

**Table 4:** Predictors of coronary heart disease among BRFSS respondents with and without obesity (BMI ≥ 30 kg/m^2^) in North Dakota (2017, 2019, and 2021).

Characteristics	Categories	BMI^1^ < 30 (n = 11,071)		BMI^1^ ≥ 30 (n = 6,021)	
		Adjusted Odds Ratio	*p*-value	Adjusted Odds Ratio	*p*-value
		(95% CI^2^)		(95% CI^2^)	
Sex	Female	0.51 (0.40, 0.66)	< 0.001	0.52 (0.38, 0.70)	< 0.001
	Male	Reference		Reference	
Age			< 0.001		< 0.001
	65+	9.49 (3.81, 23.62)	< 0.001	5.42 (2.02, 14.53)	< 0.001
	45–64	3.16 (1.28, 7.80)	0.013	2.43 (0.92, 6.40)	0.072
	18–44	Reference		Reference	
General Health	Poor/Fair	2.98 (2.29, 3.89)	< 0.001	2.34 (1.66, 3.29)	< 0.001
	Good	Reference		Reference	
Personal Doctor	Yes	1.91 (1.13, 3.23)	0.016		
	No	Reference			
High Blood Pressure	Yes	1.96 (1.48, 2.60)	< 0.001	3.35 (2.26, 4.96)	<0.001
	No	Reference		Reference	
High Cholesterol	Yes	1.68 (1.29, 2.18)	< 0.001	2.27 (1.62, 3.17)	< 0.001
	No	Reference		Reference	
Arthritis	Yes	1.72 (1.34, 2.22)	< 0.001		
	No	Reference			
COPD^3^	Yes	1.97 (1.42, 2.73)	< 0.001	2.39 (1.42, 4.02)	0.001
	No	Reference		Reference	
Diabetes	Yes	1.58 (1.15, 2.18)	0.005	1.91 (1.36, 2.69)	< 0.001
	No	Reference		Reference	

^1^Body mass index,

^2^Confidence interval,

^3^Chronic obstructive pulmonary disease

Compared to those who perceived their general health as good, those who reported fair or poor general health had significantly higher odds of CHD among respondents with obesity (OR = 2.34, 95% CI: 1.66–3.29) as well as those without obesity (OR = 2.98, 95% CI: 2.29–3.89). A number of comorbidities were associated with the odds of CHD. Hypertension (OR = 1.96, 95% CI: 1.48–2.60), high cholesterol (OR = 1.69, 95% CI: 1.29–2.18), and diabetes (OR = 1.58, 95% CI: 1.15–2.18) were associated with significantly higher odds of CHD among individuals without obesity. Similar findings were identified for those with obesity (OR [hypertension] = 3.35, 95% CI: 2.26–4.96); OR [high cholesterol] = 2.27, 95% CI: 1.62–3.17; OR [diabetes] = 1.91, 95% CI: 1.91–2.69). Having COPD was also associated with higher odds of CHD among respondents with obesity (OR = 2.39, 95% CI: 1.42–4.02) as well as those without obesity (OR = 1.97, 95% CI: 1.42–2.73).

Although having a personal doctor (OR = 1.91, 95% CI: 1.13–3.23) and arthritis (OR = 1.72, 95% CI: 1.34–2.22) were both significant predictors of coronary heart disease among respondents without obesity, they were not significantly associated with CHD among those with obesity and were therefore excluded from the final model for this group (Table 4). Prior to removal from the model in the manual backward elimination process, the odds ratios for arthritis (OR = 1.22, 95% CI: 0.88–1.69) and having a personal doctor (OR = 1.56, 95% CI: 0.81–3.03) were not significantly different from 1 (*p =* 0.227 and *p* = 0.187, respectively). There was no evidence of confounding or interaction in either of the multivariable models. Results of the Hosmer-Lemeshow goodness-of-fit test for both models did not indicate lack of fit to the data (χ^2^ = 10.81, *p* = 0.2126 and χ^2^ = 6.99, *p* = 0.5377).

## Discussion

This study investigated the prevalence and predictors of coronary heart disease among adults with and without obesity in North Dakota. The overall prevalence of CHD in North Dakota (3.6%) was similar to that of neighboring states in the Midwest and Western US (South Dakota, Nebraska, Montana and Minnesota), and was slightly lower than the national average (4.4%) in 2022 [30]. North Dakota differs from states with relatively high prevalence of CHD, such as those in the Southeastern US [30], with respect to socioeconomic and demographic characteristics. For instance, North Dakota’s population has a lower proportion of Black or African American residents, higher median household income, and lower levels of poverty [31]. While the prevalence of CHD in North Dakota has exhibited mild fluctuations over the years [30], heart disease remains the leading cause of death in the state [8]. Coronary heart disease prevalence was significantly higher among respondents with obesity compared to those without obesity in this study, consistent with findings of previous research, which has identified obesity as a key risk factor for coronary heart disease [13, 32].

Many of the characteristics associated with the odds of CHD in the current study were shared between those with and without obesity. For instance, the odds of CHD among female respondents were significantly lower than those of male respondents regardless of whether a respondent had obesity. This is consistent with findings of previous research; on average, women are diagnosed with CHD ten years later than men [33]. Although the underlying mechanisms of biological sex-related differences in the occurrence of cardiovascular disease have not been fully characterized [34], the higher prevalence of CHD observed among male respondents in this study may also reflect differences in psychosocial characteristics and/or risk behaviors such as smoking, which is more common among men than women [34, 35]. To some extent, lower prevalence of heart disease in women could also reflect underdiagnosis of the disease [36].

High blood pressure is a well-known risk factor for coronary heart disease, and was associated with increased odds of CHD among survey respondents with and without obesity in this study. Hypertension results in endothelial injury and promotes atherosclerosis, and can directly lead to angina and myocardial ischemia [37], and has consistently been identified as a leading cause of CHD [38–40]. Managing blood pressure with lifestyle changes, medication, and regular monitoring is critical for lowering the risk of CHD and its consequences, such as angina and heart attack [37]. High cholesterol was also associated with significantly higher odds of CHD regardless of whether a respondent had obesity, a finding that is consistent with previous research and highlights the importance of identifying and managing hypercholesterolemia [41, 42]. Indeed, more than 1 in 4 North Dakota adults have been diagnosed with high cholesterol [43]. Elevated cholesterol levels contribute to atherosclerotic plaque development and subsequent arterial blockages, which can result in heart disease [44–46]. The observed association between diabetes and CHD among North Dakota adults is also consistent with previous research, which has shown that both type 1 and type 2 diabetes are related to a significant increase in coronary heart disease risk due to multivessel atherosclerosis [47]. In addition to being a risk factor for CHD, diabetes is associated with an increased risk of mortality among individuals with CHD [48]. Chronic obstructive pulmonary disease was another condition significantly associated with coronary heart disease among North Dakota adults, regardless of whether a respondent had obesity. Epidemiological evidence has previously linked COPD to coronary heart disease risk [47] as well as increased cardiovascular mortality [49].

In general, findings of this study indicated that having one or more other chronic diseases, namely hypertension, high cholesterol, diabetes, and/or COPD, was associated with increased odds of coronary heart disease among North Dakota adults regardless of whether a respondent had obesity. While the magnitudes of association between these conditions and CHD tended to be slightly higher in the model for those with obesity compared to those without obesity, the 95% confidence intervals for their odds ratios overlapped, indicating these differences were not statistically significant. Regardless, the co-occurrence of CHD and other chronic illnesses observed among North Dakota adults in the present study highlights the challenge of managing multiple chronic conditions, and has implications for the control of coronary heart disease as well as other non-communicable diseases [50]. There is a growing body of evidence indicating that the presence of one chronic illness increases the risk of developing others [51]. Furthermore, there is a high prevalence of multimorbidity among U.S. adults, approximately 40% of whom have been diagnosed with multiple chronic conditions [52]. This has primarily been attributed to aging of the population and risk behaviors such as poor dietary habits, lack of exercise, and tobacco use [52]. Control measures that promote prevention, prompt diagnosis and management of high blood pressure, high cholesterol, COPD and diabetes will be important for reducing the burden of CHD in North Dakota.

The observed association between older age and the odds of CHD in this study is also an expected finding, as age is a major known risk factor for cardiovascular diseases [53–55]. Among respondents without obesity, significantly higher odds of CHD were observed among individuals aged 45–64 years and those aged 65 and above compared to those between the ages of 18 and 44. Among those with obesity, individuals aged 65 years and older had higher odds of CHD compared to those between 18 and 44. It is worth noting, however, that although middle age (45–64 years) was associated with higher odds of CHD among those without obesity, it was not a significant predictor of CHD among those with obesity after accounting for other comorbidities and perceived overall health status. Self-reported general health was also associated with coronary heart disease regardless of obesity status, with higher odds of CHD among respondents who perceived their general health to be fair or poor. Due to the cross-sectional design of this study, it is not possible to determine causality, although the observed association may reflect a bidirectional relationship. For instance, perceived health status may have been influenced by the presence of coronary heart disease due to impacts of CHD on physical functioning, quality of life, and overall general health [56–58]. On the other hand, poor general health may precede the development of coronary heart disease, because those who perceive their health as poor may have risk factors for CHD including socioeconomic risk factors (e.g. low levels of income and educational attainment), health behaviors such as smoking, and/or other chronic conditions (e.g. overweight, hypertension, high cholesterol, diabetes and asthma) [59–62].

Interestingly, the current study identified two variables that only had statistically significant associations with coronary heart disease among respondents without obesity: self-reported arthritis and having a personal doctor. Arthritis is a group of chronic inflammatory conditions, and chronic inflammation and the use of analgesic medications (e.g. non-steroidal anti-inflammatory drugs) are both associated with higher risk of cardiovascular disease [63–65]. The two most common types of arthritis among adults in the US are osteoarthritis and rheumatoid arthritis [66]. It is important to note that in the BRFSS, the presence of arthritis is based upon whether a respondent reports a diagnosis of arthritis by a healthcare professional, and there is no distinction between different types of arthritis. In contrast with our findings among those with obesity, previous research reported that osteoarthritis was associated with increased odds of self-reported heart disease after controlling for BMI [67]. Further investigation may be warranted to determine why arthritis was not significantly associated with coronary heart disease among this group of respondents. Among those without obesity, the observed association between arthritis and coronary heart disease could, to some extent, be attributable to decreased physical activity [68], a known risk factor for both arthritis and coronary heart disease. Arthritis itself is an important cause of chronic pain and activity limitations among US adults [69, 70]. It is possible that arthritis is less of an important determinant of physical activity among individuals who have obesity than those without obesity. However, while physical activity was associated with reduced odds of CHD in univariable models, it was not a significant predictor in the final multivariable models after accounting for age, sex, other chronic conditions and perceived general health status. It is also worth noting that many patients with rheumatoid arthritis are affected by loss of lean body mass, and have a higher relative percentage of body fat than individuals of equal weight who do not have arthritis [65, 71, 72]. The difference in body fat percentage between individuals with rheumatoid arthritis and the general population is largest for those in the “healthy weight” and “underweight” ranges for BMI [71]. This may have contributed to the observed association between arthritis and CHD among those without obesity but not those with obesity in the present study. These findings highlight the importance of screening for cardiovascular disease among those with arthritis, including those who may not have other “classical” risk factors such as obesity [73].

Similarly, while having a personal doctor was associated with higher odds of CHD among respondents without obesity, no such association was observed among those with obesity. Respondents with obesity who did not have CHD may receive regular medical care for other conditions, which could explain the observed lack of association between CHD and having a personal doctor in the model for respondents with obesity. Obesity is an important risk factor for a number of chronic illnesses [2], and respondents with obesity had significantly higher prevalence of hypertension, diabetes, and high cholesterol than those without obesity in the current study. In addition, a significantly higher percentage of respondents with obesity reported having a personal doctor compared to those without obesity. Thus, the lack of association between CHD and having a regular source of care among respondents with obesity in the current study may reflect the higher prevalence of comorbidities in this group. In contrast, among those without obesity, who tended to have fewer comorbidities overall, those who have been diagnosed with CHD may be more likely to seek regular medical care. Alternatively, the observed association among those without obesity could suggest that individuals with a regular source of medical care are more likely to receive diagnostic testing for CHD than those without a personal doctor. This would be consistent with the lead time bias proposed to contribute to the obesity paradox, in which individuals with a BMI within the normal range are less likely to receive diagnostic testing for CHD, are diagnosed at a more advanced stage of disease, and therefore have a worse prognosis [17].

### Strengths and limitations

To the authors’ knowledge, this is the first study to investigate and compare predictors of coronary heart disease among adults with and without obesity in North Dakota. Stratification of the analysis based upon the presence of obesity permitted the identification of predictors that differed between the two groups of respondents. Strengths of this study included a relatively large sample size, and the quality of BRFSS data with respect to reliability and validity [74]. In addition, sampling methods of the BRFSS survey result in estimates that are representative of the adult population in North Dakota, which is useful to inform health planning efforts in the state. However, this study was not without limitations. Key demographic variables, such as income, educational attainment, marital status and urban-rural location were not available in the dataset. Furthermore, while data from 2017–2021 were available for this study, some variables of interest (arthritis, high blood, pressure, high cholesterol, fruit and vegetable consumption) were not available in the 2018 and 2020 datasets, which were therefore excluded from the study. In addition, BRFSS survey data are self-reported, and can therefore be liable to recall bias and social desirability effects. Furthermore, due to the cross-sectional nature of the BRFSS survey, the temporal relationships between respondent characteristics and the development of CHD are unknown. Therefore, it is important to note that while this study provides evidence of association between CHD and variables identified as significant predictors in the logistic regression model, it does not provide evidence of causality. Despite the above limitations, findings of this study provide important information on the determinants of coronary heart disease among adults with obesity and without obesity in North Dakota.

## Conclusions

This study provides information on the frequency and determinants of coronary heart disease among North Dakota adults. While the prevalence of CHD was significantly greater among adults with obesity than among those without obesity, the findings of the stratified analysis indicated that predictors of CHD for the two groups tended to be similar. The study identified a significant sex difference, with males having higher odds of CHD than females. Furthermore, the study highlights the relevance of other chronic conditions (high blood pressure, high cholesterol, chronic obstructive pulmonary disease, and diabetes), perceived general health status, and age as predictors of CHD among individuals regardless of obesity status. While respondents with obesity and without obesity shared many of the same predictors of coronary heart disease, having a personal doctor and having a diagnosis of arthritis were only associated with CHD among respondents without obesity. This study provides useful information to guide health planning and control programs that aim to reduce the burden of coronary heart disease in North Dakota.
